# Magnitude and Predictors of Subclinical Hypothyroidism in Individuals With Impaired Glucose Tolerance

**DOI:** 10.7759/cureus.83948

**Published:** 2025-05-12

**Authors:** Tejaswita Chourey, Yasmee Khan, Abhijit P Pakhare, Abhishek Singhai, Rachna Parashar, Rajnish Joshi, Mahadev Meena

**Affiliations:** 1 Internal Medicine, All India Institute of Medical Sciences (AIIMS) Bhopal, Bhopal, IND; 2 Community and Family Medicine, All India Institute of Medical Sciences (AIIMS) Bhopal, Bhopal, IND; 3 Physiology, All India Institute of Medical Sciences (AIIMS) Bhopal, Bhopal, IND

**Keywords:** glycated hemoglobin (hba1c), impaired glucose tolerance, subclinical hypothyroidism (sch), thyroid function screening, thyroid-stimulating hormone (tsh)

## Abstract

Background: The screening for thyroid function abnormalities in asymptomatic individuals remains a topic of debate. The impact of subclinical hypothyroidism (SCH) on glucose metabolism has been less extensively researched. Our study aimed to assess the feasibility of thyroid-stimulating hormone (TSH)-based screening and evaluate the prevalence of SCH among individuals with impaired glucose tolerance (IGT) in a community-based setting.

Methodology: We performed a longitudinal study in individuals with IGT by measuring clinical parameters and TSH at baseline and follow-up measurements at 6 and 12 months.

Results: We included 148 participants with IGT. The baseline mean HbA1c of the participants was 6.0% ± 0.2%, and the TSH level was 3.2 ± 2.1 µIU/mL. In the study, we found that 18 (12.2%) individuals with IGT had baseline TSH values in the SCH range (TSH 5-10 µIU/mL). Individuals with IGT who also had a TSH abnormality had significantly lower HbA1c levels as compared to those who were euthyroid (5.9% ± 0.2% vs. 6.1% ± 0.2%, *P *< 0.008). Other measurements were similar between the two groups. There was a significant decline in mean HbA1c levels on follow-up (baseline HbA1c 6.1% ± 0.2% vs. follow-up HbA1c 5.4% ± 0.7%; *P *< 0.001). One year later, 14 individuals (87.5%) with SCH reverted to normal TSH levels without specific thyroxine therapy, while 17 individuals (15.8%) who were initially euthyroid developed TSH elevations into the SCH range, suggesting dynamic fluctuations in TSH levels among individuals. There was, however, no change in mean TSH levels on follow-up (baseline 3.2 ± 1.8 µIU/mL vs. follow-up 2.9 ± 2.3 µIU/mL; *P *= 0.22). The overall prevalence of SCH at baseline and follow-up was similar (16, 13.2%, vs. 18, 14.9%; *P *= 0.855).

Conclusions: The study highlights that SCH is a transient condition among individuals with IGT. The majority of these individuals reverted to normal thyroid function after one year without requiring thyroid hormone therapy, underscoring the unstable nature of SCH.

## Introduction

Impaired glucose tolerance (IGT) is a condition where blood sugar levels are elevated beyond the normal range yet not high enough to be classified as diabetes. It represents an intermediate stage of hyperglycemia and is often viewed as a pre-diabetic condition [1]. On the other hand, subclinical hypothyroidism (SCH) is a mild form of underactive thyroid function, characterized by the thyroid gland's insufficient production of thyroid hormones. However, this production is not low enough to trigger noticeable symptoms of hypothyroidism. SCH is marked by elevated levels of thyroid-stimulating hormone (TSH), while the levels of the thyroid hormones thyroxine (T4) and triiodothyronine (T3) remain within normal reference ranges [2].

The screening for thyroid function abnormalities in asymptomatic individuals remains a topic of debate. The American Thyroid Association recommended in 2000 and 2004 that all adults over the age of 35 should undergo screening for thyroid disease by TSH levels every five years [3]. Other organizations, like the American Academy of Family Physicians, advise screening after age 60. According to the British Thyroid Association's statement in 2006, TSH levels should be retested after three to six months if screening is conducted. Additionally, there are specific recommendations for screening thyroid disorders in individuals with Type 1 diabetes mellitus (T1DM), while the rationale for TSH-based screening in individuals with Type 2 diabetes mellitus (T2DM) remains unclear [4]. The impact of SCH on glucose metabolism is likely to be subtle and has been less extensively researched. Our study aimed to assess the feasibility of TSH-based screening and to evaluate the prevalence of SCH among individuals with IGT in a community-based setting. Additionally, we sought to determine whether adults with IGT who were classified as having SCH at baseline exhibited a higher risk of worsening glycemic parameters over one year of follow-up compared to those without SCH. We also aimed to identify specific risk factors such as body mass index (BMI), waist circumference (WC), waist-to-hip ratio (WHR), and blood pressure that overlap between SCH and IGT.

## Materials and methods

Design and ethics

We performed a longitudinal study in the urban slum community of Bhopal. The study design was approved by the Institutional Human Ethics Committee for Student Research at AIIMS Bhopal (LoP number AIIMS/BPL/IHECSR/July/22/PG/06, dated February 23, 2023). All study participants provided written informed consent; only eligible and consenting individuals were included.

Setting

The study was conducted at the All India Institute of Medical Sciences, Bhopal, and associated community-based outreach services in low-income urban slum communities of Sai Baba Nagar in the city of Bhopal.

Participants

We performed a two-step screening to identify participants for our study. In the first step, we obtained home-based screening lists as provided by the Accredited Social Health Activist (ASHA) to identify individuals with random capillary blood glucose (CBG) of 100mg/dL or more. All the individuals who screened positive in this first step were given an appointment to visit the Urban Primary Health Centre (UPHC) on prespecified days of the week in a fasting state. In the second step, all such individuals were further evaluated by fasting CBG and a point-of-care HbA1c test at UPHC.

We included participants (aged >18 years) who presented to outpatient department (OPD) settings associated with AIIMS Bhopal and were diagnosed with IGT, impaired fasting glucose (fasting CBG between 100 and 125 mg/dL), and impaired HbA1c levels (HbA1c between 5.7% and 6.4%). We excluded individuals from the study who had overt hypothyroidism or a past history of thyroid disease, were already receiving thyroid hormone-modifying medications (such as thyroxine or anti-thyroid drugs), were on glucose-lowering medications, were taking concomitant steroids, were pregnant, or had known comorbidities that limited their ability to engage in effective lifestyle measures, including chronic kidney disease (CKD), heart failure with reduced ejection fraction (HFrEF), chronic obstructive pulmonary disease (COPD), chronic liver disease, or solid organ or hematological malignancies. 

Sample size estimation

Previous studies estimate that the proportion of individuals with SH among those with IGT varies from 9% (China) and 27% (India). We used the higher prevalence value of 27% and a 5% margin of error to estimate the sample size. Our estimated sample size was 303. We used the following formula for this estimation, where *n* is the sample size, *z* is the *z*-score, ε is the margin of error, and *p* is the population proportion: *n *= [*z*^2^ × *p *(1 - *p*)] ÷ ε^2^.

Given the potential loss of information and loss to follow-up, we planned to sample an additional 10% of the estimated sample size, yielding a target sample size of 330 participants at a 95% confidence level.

Study procedures

A structured interview was conducted at baseline to gather information on current symptoms, known comorbidities from the participants. Clinical measurements included BMI = weight (kg)/height (cm)^2^, WC, WHR, and blood pressure assessment. Blood glucose levels were determined using a glucometer. Point-of-care serum TSH testing and HbA1c analysis were performed on collected blood samples using the SD Biosensor F2400 analyzer (SD Diagnostics, Seoul, South Korea). Clinical consultations were provided on the same day as the baseline assessment. Follow-up appointments were scheduled at six and 12 months, during which participants were invited to return by phone call to the UPHC for evaluation.

Study outcomes

The key study outcome was glycemic and thyroid status at follow-up. Glycemic status was defined as overt diabetes mellitus (DM) if the follow-up HbA1c level was greater than 6.4%, and as IGT if HbA1c was between 5.7% and 6.4%. Since the upper limit of serum TSH levels was generally 4.0-5.0 µIU/mL in most facilities, we defined SCH in our study as a TSH range greater than 5.0 and up to 10.0 µIU/mL. For feasibility outcomes, we recorded the refusals for intravenous blood sampling, failure events for obtaining an adequate sample, and equipment failure events. We estimated the ratio of the number of test kits used and reported tests to assess the efficiency of point-of-care TSH and HbA1c testing.

Statistical analysis

We collected primary data in a standardized form and entered the data in MS Excel. We conducted a descriptive analysis and described the variables concerning measures of central tendency and distribution (e.g., mean [standard deviation], median [Range], and frequency [proportions]). We compared the distribution using a *t*-test for means, a Wilcoxon rank-sum test for medians, and a chi-square test for frequency. Based on the study outcomes, we conducted baseline and end-line comparisons of various parameters within participant subgroups. We also performed multivariable regression analysis to evaluate for independent predictors of SCH among individuals with IGT. All statistical analysis was done using the statistical software R. We considered a *P*-value of <0.05 as significant.

## Results

**Figure 1:**
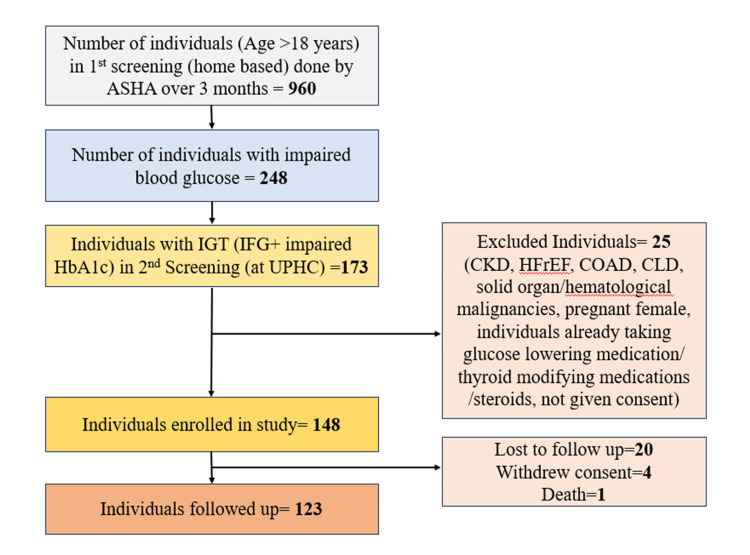
Study flow diagram. IGT, impaired glucose tolerance; IFG, impaired fasting glucose; UPHC, Urban Primary Health Care Centre; CKD, chronic kidney disease; HFrEF, heart failure with reduced ejection fraction; COAD, chronic obstructive airway disease; CLD, chronic liver disease

Between April and June 2023, a total of 960 individuals were screened by the ASHA in urban slum areas. Of these, 248 were found to have impaired blood sugar and were referred to UPHC for a second screening. Among those referred, 173 met the inclusion criteria, as their glycemic parameters (FCG and HbA1c levels) were in the IGT range. Of these, 148 were included in the study, while the remaining 25 were excluded (Figure 1).

**Figure 2:**
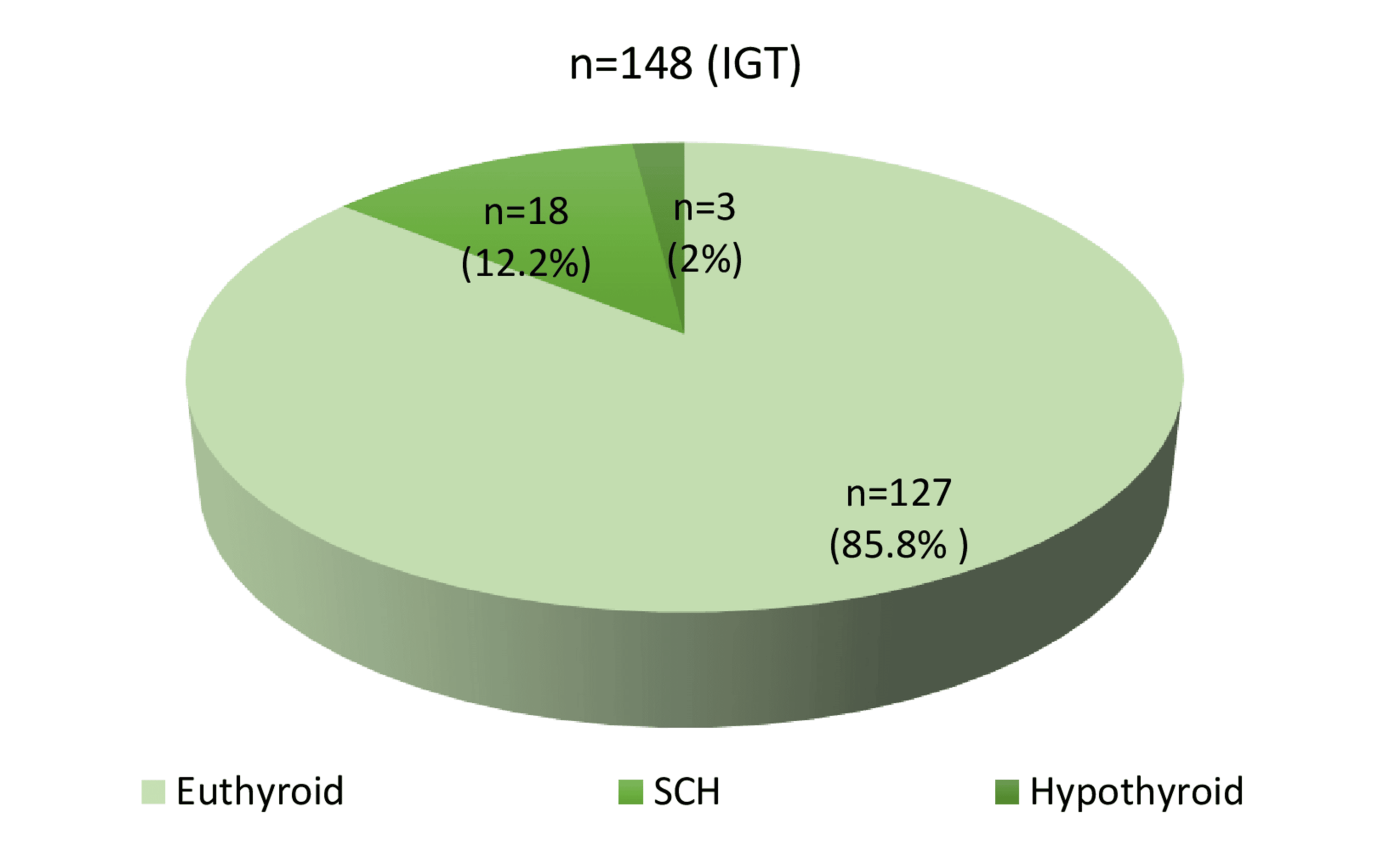
Thyroid abnormalities based on TSH screening in individuals with IGT (n = 148). TSH, thyroid-stimulating hormone; IGT, impaired glucose tolerance

The included participants (*n* = 148) were middle-aged (mean age 47.8 ± 12.0 years), and most of them were women (85, 57.4%). Of the 148 individuals with IGT, 18 (12.2%) had TSH levels between 5 and 10 µIU/mL, so we classified them as having SCH. An additional 3 (2%) individuals had a TSH level above 10 µIU/mL, classified as hypothyroid. The remaining 127 (85.8%) were euthyroid (Figure 2).

The baseline mean HbA1c of the participants was 6.0% ± 0.2%, and the TSH level was 3.2 ± 2.1 µIU/mL. The distribution of participants across the euthyroid (*n* = 127) and thyroid dysfunction (*n* = 21) categories suggests that a significantly higher proportion of participants with thyroid dysfunction had known hypertension, with a significant *P*-value of <0.05 (38.1% vs. 14.2%, *P* = 0.013). Individuals with IGT who also had a TSH abnormality had significantly lower HbA1c levels as compared to those who were euthyroid (5.9% ± 0.2% vs. 6.1% ± 0.2%, *P *< 0.008). Other parameters were similar across the two groups (Table 1).

**Table 1:** Distribution of baseline variables across thyroid dysfunction categories (TSH < 5, TSH > 5). Test statistic: *t* = *t*-test, *χ*² = chi-square. HTN, hypertension; HbA1c, hemoglobin A1c; WC, waist circumference; WHR, waist-to-hip ratio; BMI, body mass index; SBP, systolic blood pressure; DBP, diastolic blood pressure; TSH, thyroid-stimulating hormone

Characteristic	TSH < 5, *n* = 127, mean ± SD or *n* (%)	TSH > 5, *n* = 21, mean ± SD or *n* (%)	Test statistics	*P*-value
Age (years)	47.528 ± 11.977	49.667 ± 12.391	*t* = -0.805	0.422
Sex (Female)	71 (55.9%)	14 (66.7%)	*χ*² = 0.856	0.356
Sex (Male)	56 (44.1%)	7 (33.3%)		
HTN	18 (14.2%)	8 (38.1%)	*χ*² = 6.163	0.013
Baseline HbA1c	6.071 ± 0.246	5.924 ± 0.195	*t* = 2.713	0.008
Baseline weight	60.846 ± 13.719	59.762 ± 8.899	*t* = 0.213	0.832
Baseline height	155.992 ± 7.097	154.667 ± 5.994	*t* = 0.826	0.411
Baseline WC	94.457 ± 13.790	97.190 ± 12.077	*t* = -0.880	0.38
Baseline WHR	1.015 ± 0.116	1.032 ± 0.094	*t* = -0.440	0.66
Baseline BMI	25.008 ± 5.402	25.031 ± 3.764	*t* = -0.637	0.526
Baseline SBP	126.969 ± 15.495	135.810 ± 22.873	*t* = -1.342	0.18
Baseline DBP	80.079 ± 8.964	80.810 ± 11.007	*t* = -0.001	>0.999

We followed 123 participants with repeat measurements. Compared to baseline, there was a significant decline in mean HbA1c levels at follow-up (baseline: 6.1% ± 0.2% vs. follow-up: 5.4% ± 0.7%; *P* < 0.001). However, there was no significant change in mean TSH levels (baseline: 3.2 ± 1.8 µIU/mL vs. follow-up: 2.9 ± 2.3 µIU/mL; *P* = 0.22). There were no notable changes in the other parameters, except for diastolic blood pressure (Table 2).

**Table 2:** Comparison of characteristics at baseline and 12-month follow-up (n = 123). Test statistics: *t* = *t*-test, *χ*² = chi-square HbA1c, hemoglobin A1c; TSH, thyroid-stimulating hormone; Weight, weight (kg); Height, height (cm); WC, waist circumference; BMI, body mass index; SBP, systolic blood pressure; DBP, diastolic blood pressure; WHR, waist-to-hip ratio; TSH, thyroid-stimulating hormone

Characteristic	Baseline, *n* = 123, mean ± SD, n (%)	Follow-up, n = 123, mean ± SD, n (%)	Difference	95% CI	Test statistics	P-value
HbA1c	6.061 ± 0.242	5.362 ± 0.741	0.7	0.56, 0.84	t = 6.98	<0.001
TSH	3.167 ± 1.813	2.858 ± 2.295	0.31	-0.19, 0.81	t = 1.23	0.221
Weight	61.260 ± 13.790	60.719 ± 13.631	0.54	-0.28, 1.4	*t* = 1.30	0.194
Height	155.661 ± 7.075	155.694 ± 7.059	-0.03	-0.68, 0.61	*t* = -0.10	0.92
WC	95.289 ± 13.778	94.905 ± 13.474	0.38	-0.53, 1.3	*t* = 0.83	0.407
BMI	25.291 ± 5.461	25.057 ± 5.390	0.23	-0.20, 0.67	*t* = 1.06	0.292
SBP	128.463 ± 16.570	129.612 ± 10.863	-1.1	-4.2, 1.9	*t* = -0.75	0.457
DBP	79.901 ± 9.003	83.347 ± 7.315	-3.4	-5.3, -1.5	*t* = -3.92	<0.001
WHR	1.016 ± 0.114	1.012 ± 0.104	0	-0.01, 0.01	*t* = 0.76	0.447
TSH <5	105 (86.8%)	103 (85.1%)	-	-	χ² = 0.34	0.855
TSH >5	16 (13.2%)	18 (14.9%)	-	-		

Of the 18 individuals who had SCH at baseline, follow-up values were available for 16. Of these, only 2 (12.5%) had a persistent SCH. All the other 14 (87.5%) had their TSH values reverted to less than 5 µIU/mL. None of these individuals were prescribed thyroxine. On the other hand, of the 107 individuals with their TSH levels below 5 µIU/mL, 17 (15.8%) had a rise in TSH to greater than 5 µIU/mL on follow-up. While the overall prevalence of SCH at baseline and follow-up was similar (16, 13.2% vs. 18, 14.9%; *P *= 0.855), there was a bidirectional change in the TSH status.

## Discussion

Our study found that among individuals with IGT, about 18 (12.2%) had their TSH values in the SCH range. One year later, nearly 90% had their values reverted to normal without any specific thyroxine therapy. The conversion of TSH values from a euthyroid to SCH range over one year was of similar magnitude. This suggests that SCH is an unstable or transient metabolic state. Further, those individuals with abnormal TSH values had no adverse glycemic status. Individuals with IGT who had a worsening of their glycemic status had an improvement in their TSH values. While measuring TSH values in a community-based setting was feasible, this additional screening does not seem to add value to overall clinical management. 

Some studies explored various aspects of thyroid dysfunction, particularly SCH, in different populations. Fade et al. studied 1,210 elderly patients, finding a higher prevalence of thyroid dysfunction in females, with 17.8% progressing to hypothyroidism over 12 months, supporting the recommendation for routine thyroid screening in those over 60 due to high dysfunction rate [5]. Li et al. followed 505 Chinese patients with mild SCH for three years and found that 43.8% had persistent SCH, while 49.7% reverted to normal thyroid function. Thyroid peroxidase (TPO) antibodies and elevated cholesterol were identified as key predictors of progression to overt hypothyroidism. These findings highlight the need for individualized monitoring of TSH and cholesterol levels in patients with mild SCH [6]. Somwaru et al., studying an elderly cohort, found that 56% of SCH cases persisted over four years, with higher TSH levels (≥10 µIU/mL) predicting progression. Across studies, TSH levels, TPO antibodies, and cholesterol were important predictors [7]. Hence, careful clinical analysis combined with markers such as TSH levels, thyroid antibodies (e.g., TPO antibodies), and ultrasonography, as highlighted in population-based cohort studies, can guide clinicians in deciding whether to treat SCH. In individuals over 60 years, diagnosing SCH becomes more challenging due to age-related increases in TSH levels, requiring a cautious interpretation of these values. Following up with thyroid antibodies and ultrasonography, which can detect autoimmune thyroiditis through hypo-echogenicity in the thyroid, significantly improves diagnostic accuracy in these cases [8]. In the treatment of SCH with levothyroxine (LT4), there is a risk of cardiovascular events due to overdose. LT4 is generally not recommended for patients with serum TSH levels below 10 µIU/mL unless symptoms of hypothyroidism are present. However, for patients under 70 years old with TSH levels between 7 and 10 µIU/mL, LT4 may be considered depending on individual risk factors such as cardiovascular disease. The long-term benefits of LT4 on symptoms and cardiovascular outcomes in SCH remain unproven, and guidelines, such as the 2019 British Clinical Practice Recommendations, controversially advise against LT4 for most patients, except in certain cases like pregnancy or severe symptoms [9]. 

It is essential not to alarm the family when SCH is detected because this is a biochemical situation that normalizes in most cases. Thyroid hormone therapy was not associated with improvements in quality of life or thyroid-related symptoms in adults with SCH [10]. This fact and the mean cost of specialized care suggest that the first step should be repeated TSH measurements in primary care [11]. According to the Royal College of Physicians, United Kingdom (RCP), screening for thyroid dysfunction is unjustified in the general population, but it recommends screening in elderly females [12]. This underscores the need for a personalized approach to SCH management, balancing the potential benefits of treatment with the risks of unnecessary intervention.

The study exhibits several strengths, designed as a longitudinal study, which allowed for observing changes over time, providing insights into the dynamic relationship between IGT and SCH. Conducted in the community, adding value to the study’s external validity. Using ASHAs and UPHCs for initial screenings made the study more accessible to participants and ensured integration with existing healthcare services. Additionally, the two-step screening process using home-based listings and subsequent UPHC-based fasting glucose and HbA1c tests offered a robust mechanism for identifying participants with IGT. The study has some limitations that may have influenced the results. Our study did not include clinical outcome measures such as symptom burden or cardiovascular risk markers, which are important for evaluating the broader implications of SCH. While the tests conducted were logistically feasible, they were not ideal for classifying IGT or SCH. For example, oral glucose tolerance tests (OGTT), T4 as well as T3 levels, which are essential for precisely classifying these conditions, were not performed due to resource limitations in a community setting. HbA1c alone may misclassify some individuals, especially in populations with anemia or hemoglobinopathies. Anti-TPO antibody testing, which is important for distinguishing transient SCH from early autoimmune thyroid disease, was also not performed. One of the limitations of this study is its reduced statistical power. A post hoc power analysis indicated that, given the observed sample size and expected proportions, the power was approximately 50%, substantially lower than the intended 80% due to logistical constraints. This reduction increases the risk of a Type II error, failing to detect a true association or difference if one exists. There was variability in the time intervals between initial and subsequent HbA1c and TSH measurements, which could have introduced bias into the findings. Furthermore, other key metabolic parameters, such as lipid profiles and liver function tests (AST/ALT), were not measured, limiting the comprehensive assessment of participants' metabolic health. Additionally, while a long-term study is necessary to better understand the correlation between IGT and SCH, we were able to follow up with most participants only once, limiting the ability to observe long-term trends.

## Conclusions

This study highlights that SCH is a transient condition among individuals with IGT. Interestingly, the majority of these individuals reverted to normal thyroid function after one year without requiring thyroid hormone therapy, underscoring the unstable nature of SCH. The study found no adverse glycemic impact among individuals with thyroid dysfunction. Those with abnormal TSH values had slightly better glycemic outcomes. Although screening for thyroid dysfunction in a community setting was feasible, the results suggest that such screening may not significantly influence clinical management without specific symptoms. This aligns with the broader literature, which suggests that routine treatment of mild SCH may offer limited benefit, especially in individuals without cardiovascular risk factors. Therefore, an individualized approach to thyroid screening and management remains the most prudent, particularly in asymptomatic individuals. However, a study with a larger sample size is needed to draw definitive conclusions, as the present study was underpowered due to logistical constraints and a relatively small SCH subgroup.
